# Comparison of static immersion and intravenous injection systems for exposure of zebrafish embryos to the natural pathogen *Edwardsiella tarda*

**DOI:** 10.1186/1471-2172-12-58

**Published:** 2011-10-17

**Authors:** Joost J van Soest, Oliver W Stockhammer, Anita Ordas, Guido V Bloemberg, Herman P Spaink, Annemarie H Meijer

**Affiliations:** 1 Institute of Biology, Leiden University, PO Box 9502, 2300 RA Leiden, The Netherlands

## Abstract

**Background:**

The zebrafish embryo is an important *in vivo *model to study the host innate immune response towards microbial infection. In most zebrafish infectious disease models, infection is achieved by micro-injection of bacteria into the embryo. Alternatively, *Edwardsiella tarda*, a natural fish pathogen, has been used to treat embryos by static immersion. In this study we used transcriptome profiling and quantitative RT-PCR to analyze the immune response induced by *E. tarda *immersion and injection.

**Results:**

Mortality rates after static immersion of embryos in *E. tarda *suspension varied between 25-75%, while intravenous injection of bacteria resulted in 100% mortality. Quantitative RT-PCR analysis on the level of single embryos showed that expression of the proinflammatory marker genes *il1b *and *mmp9 *was induced only in some embryos that were exposed to *E. tarda *in the immersion system, whereas intravenous injection of *E. tarda *led to *il1b *and *mmp9 *induction in all embryos. In addition, microarray expression profiles of embryos subjected to immersion or injection showed little overlap. *E. tarda*-injected embryos displayed strong induction of inflammatory and defense genes and of regulatory genes of the immune response. *E. tarda*-immersed embryos showed transient induction of the cytochrome P450 gene *cyp1a*. This gene was also induced after immersion in *Escherichia coli *and *Pseudomonas aeruginosa *suspensions, but, in contrast, was not induced upon intravenous *E. tarda *injection. One of the rare common responses in the immersion and injection systems was induction of *irg1l*, a homolog of a murine immunoresponsive gene of unknown function.

**Conclusions:**

Based on the differences in mortality rates between experiments and gene expression profiles of individual embryos we conclude that zebrafish embryos cannot be reproducibly infected by exposure to *E. tarda *in the immersion system. Induction of *il1b *and *mmp9 *was consistently observed in embryos that had been systemically infected by intravenous injection, while the early transcriptional induction of *cyp1a *and *irg1l *in the immersion system may reflect an epithelial or other tissue response towards cell membrane or other molecules that are shed or released by bacteria. Our microarray expression data provide a useful reference for future analysis of signal transduction pathways underlying the systemic innate immune response versus those underlying responses to external bacteria and secreted virulence factors and toxins.

## Background

In the last decade the zebrafish has been firmly established as a model for infectious diseases [1-4]. The increasing popularity of the zebrafish is due to its many useful characteristics. The embryos develop fast *ex utero *and are transparent, making it possible to follow infection *in vivo*. The real-time analysis of infection processes in this model is facilitated by the development of transgenic zebrafish lines with fluorescently marked immune cell populations that can be used in combination with differential fluorescently labeled pathogens [5-8]. In addition, reverse and forward mutagenesis screens are possible, as are antisense knock-down techniques using morpholinos.

Like all jawed vertebrates the zebrafish possesses an innate and adaptive immune system. Innate immunity forms the first line of defense against invading microorganisms. Humoral components of the innate immune system, such as complement and acute phase proteins, were shown to be expressed in embryos and larvae and could be induced by lipopolysaccharide (LPS) challenge or infection [9,10]. The major cell types required for cell-mediated innate immunity, macrophages and neutrophils, also develop during the first days of zebrafish embryogenesis [11-13]. An essential step in innate immunity is the recognition of invading microorganisms by pattern recognition receptor families, the most well studied being the Toll-like receptor (TLR) family. The TLRs activate a signaling pathway leading to a cytokine response and the activation of antimicrobial defense genes [14]. The TLR signaling components are highly conserved between zebrafish and humans [15,16]. In adults the innate and adaptive immune systems are tightly connected, however in the zebrafish embryo there is a temporal segregation. Whereas innate immunity is functional as early as 1 day post fertilization (dpf) [11,17,18], adaptive immunity does not reach full maturity until approximately 4 weeks post fertilization [13,19,20]. This makes the zebrafish embryo a useful *in vivo *model to study vertebrate innate immunity separate from adaptive immunity [3].

Bacterial infection models that have been developed in zebrafish differ in mode and time of infection, inoculum size, pathogenicity and host response [2-4]. The most common method of infection is injection, with the caudal vein as injection site at 1 dpf or the yolk circulation valley at 2 dpf [21]. *Salmonella typhimurium*, a mammalian pathogen, was shown to be lethal to zebrafish embryos after caudal vein injection of a low dose of 25-50 bacteria [22]. In contrast, injection of *E. coli *or an LPS-mutant of *Salmonella typhimurium *(Ra-mutant) was not lethal and the bacteria were cleared efficiently by the embryonic innate immune system [22]. *Pseudomonas aeruginosa*, a broad host range pathogen, capable of infecting plants, invertebrates, and vertebrates, was lethal after injection into the yolk circulation valley at 10-100-fold higher injection inocula than used for *S. typhimurium*, while *Burkholderia cenocepacia *was recently shown to cause a lethal infection upon intravenous injection at a dose of less than 10 bacteria [23-25]. At relatively high doses, also gram-positive bacteria such as *Streptococcus *and *Staphylococcus *species were shown to be capable of causing lethality upon injection in both adults and embryos [26-29]. Injection of embryos with *Mycobacterium marinum *does not lead to a lethal infection, but the immune system is unable to clear this bacterium, leading to a chronic infection. This chronic infection is characterized by aggregation of macrophages into granuloma-like structures similar to the tuberculous granulomas found in human tuberculosis patients [17]. The different infection models were useful to study bacterial virulence factors and the response of the host immune system [3,9,30,31].

For experimental screening, intravenous injection of zebrafish embryos is a relatively low throughput method. For high throughput analysis, such as mutant or drug screens, it is highly desirable to have an easier method of infection like static immersion. Thus far, the only bacterial pathogens that were reported to be capable of infecting zebrafish embryos without the need of injection are *Edwardsiella tarda *and *Flavobacterium columnare *[32,33], which are Gram-negative naturally occurring fish pathogens. *E. tarda *is primarily known for infecting channel catfish, Japanese eel and flounder, in which it causes edwarsiellosis, a generalized septicemia. Pressley and colleagues showed that 24 hpf zebrafish embryos immersed for five hours in a suspension of *E. tarda *had a cumulative mortality rate of 31% after 14 days, compared to 11% in the control embryos [32]. In addition, the zebrafish embryos showed peaks in the expression of *tnfa *and *il1b *at 2 and 4 hours post exposure, respectively. In adults, *E. tarda *is capable of causing infection by static immersion in combination with dermal abrasion [32].

The aim of this study was to compare the robustness of immersion and injection methods for treatment of 1-day-old zebrafish embryos with *E. tarda *and to identify marker genes that provide a reproducible read-out for the immune response. We set out with a microarray analysis of embryos subjected to immersion in *E. tarda*, and used *E. coli *and *P. aeruginosa*, both non-lethal in the immersion method, for comparison. Several markers were selected for a qPCR time-course analysis of the immersion method and for comparison with caudal vein injection. Marker expression analysis at single embryo level revealed high variation between individuals in response to static immersion. In contrast, qPCR and microarray analysis of single embryos that were systemically infected by caudal vein injection showed a consistent profile of strong activation of the proinflammatory marker genes *il1b *and *mmp9*. We conclude that the injection method is best suited for studying the innate immune response towards systemic infection, while the immersion system is useful for studying epithelial or other tissue responses towards cell membrane or other molecules that are shed or released by bacteria.

## Results

### Survival of zebrafish embryos after immersion in *E. tarda *suspension

**Figure 1:**
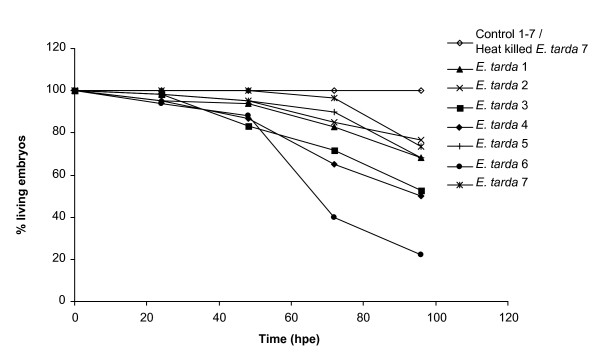
**Survival curve of zebrafish embryos treated by immersion in *E. tarda *suspension**. For each of the independent 7 experiments 20 embryos at 25 hpf were immersed for 5 h in 10^8 ^CFUs/ml of *E. tarda *or in clean egg water as a control. Subsequently embryos were washed and transferred to fresh egg water, and survival was monitored for 4 days. In one experiment, heat-killed bacteria (45 min at 95°C) were included as an extra control group. Survival varied between approximately 25 and 75%.

In order to test the *E. tarda *immersion method for future screening applications, we set out to confirm the results obtained by Pressley *et al. *[32]. To this end, zebrafish embryos at 25 hpf were immersed for 5 h in 10^8 ^CFUs/ml of *E. tarda *and survival was monitored for four days. The ability of *E. tarda *to cause mortality by static immersion was confirmed, while exposure to heat-killed bacteria did not cause mortality (figure 1). However, the percentage of mortality following *E. tarda *exposure after 4 days was found to be quite variable, ranging from 25% to 75% between different experiments (figure 1). In addition to *E. tarda*, we also tested the ability of *P. aeruginosa *to establish a lethal infection by static immersion, using strains PAO1 and PA14. However, even with concentrations up to 10^9 ^CFUs/ml, these strains were unable to cause mortality (data not shown).

### Microarray analysis of embryos subjected to the immersion system

**Figure 2:**
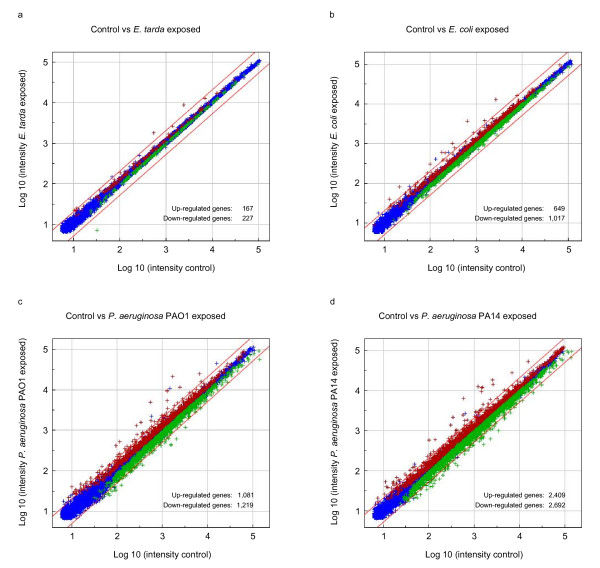
**Intensity plots from microarrays of zebrafish embryos treated by immersion in *E. tarda*, *E. coli*, or *P. aeruginosa *suspensions**. Embryos were immersed at 25 hpf in *E. tarda *(a), *E. coli *(b) and *Pseudomonas aeruginosa *PAO1 (c) and PA14 (d) suspensions, or in clean egg water as a control. RNA for microarray analysis was isolated from pools of 20 embryos at 5 h post exposure (hpe). RNA samples from embryos exposed to bacterial suspensions and control embryos were hybridized against a common reference from all treatment groups. The intensity plots show comparisons of treatment versus control groups derived from re-ratio analysis against the common reference. Significantly (P < 0.0001) up-regulated genes are shown in red, down regulated genes are shown in green and remaining genes in blue.

The variability of the mortality rate in the *E. tarda *immersion assay was high. Therefore, we performed microarray analysis on pools of 20 zebrafish embryos immersed at 25 hpf for 5 h in *E. tarda *to find markers for a reproducible readout of the immune response as alternative. To determine if we could differentiate between reactions towards pathogenic and non-pathogenic bacteria, *E. coli *DH5α and *P. aeruginosa *strains PAO1 and PA14 were tested in addition. Surprisingly, *E. tarda *immersed embryos showed the smallest signature set in terms of gene induction or repression (figure 2a). The number of differentially expressed genes after *E. coli *immersion was four times higher (figure 2b), with *P. aeruginosa *PAO1 immersion six times higher (figure 2c), and with *P. aeruginosa *PA14 immersion 13 times higher (figure 2d) (Additional file 1, Table S1).

Surprisingly, very few of the genes up-regulated in the zebrafish embryo after exposure to *E. tarda *were immune related. Although transient induction of *il1b *and *tnfa *was previously observed by Pressley et al. [32], no induction of these genes was detected in our microarray analysis. Furthermore, expression of *mmp9*, one of the most strongly induced markers after *Salmonella *infection [9], was only slightly up-regulated (1.4 times). In total only 21 genes showed 2-fold or higher levels of up-regulation (P < 1.0 E-4) after *E. tarda *exposure (Additional file 1, Table S1). Some of these genes have a possible immune-related function. The highest induced gene after exposure to *E. tarda *was *cyp1a *(9.8-fold induction), which encodes a cytochrome P450 enzyme known to be involved in the toxic response [34,35]. As shown in Additional file 1, Table S1, this gene is also highly induced after *P. aeruginosa *and *E. coli *exposure. The second highest induced gene was *zgc:154020 *(6.8-fold), which shows 62.1% identity with *immunoresponsive gene 1 *(*irg1*) from *Mus musculus*, a gene with homology to bacterial methylcitrate dehydratase, which is up-regulated in murine macrophages after exposure to LPS, cytokines, and mycobacteria [36-39]. *Zgc:154020 *will hereafter be referred to as *irg1-like *(*irg1l)*. Like *cyp1a*, *irg1l *was also highly up-regulated after *P. aeruginosa *and *E. coli *exposure. A third gene with a possible immune-related function is stanniocalcin 1 (*stc1*), which was only induced after *E. tarda *exposure (2.1-fold). Stanniocalcin is involved in Ca^2+ ^homeostasis in fish [40,41], but in humans has also been implicated in inflammatory responses [42-44].

To compare the responses of zebrafish embryos to immersion with the different bacterial strains, we performed a gene ontology analysis on all genes showing differential expression in the microarray analysis (Additional file 2, Table S2). In embryos immersed in *P. aeruginosa *PAO1 and PA14, and in *E. coli*, but not in embryos immersed in *E. tarda*, genes with the GO-term "response to stimulus" were significantly enriched. The largest group of up-regulated genes with this GO-term (61 genes) was observed in the case of immersion with *P. aeruginosa *PA14. Further analysis into the "response to stimulus" GO category revealed that in particular genes with the GO-term "response to stress" were up-regulated (41 genes in the case of PA14), while only few genes were associated with the GO-term "immune response" (6 genes in the case of PA14). An overview of the genes with the GO-term "response to stimulus" that were up-regulated in response to the different bacteria is given in Additional file 3, Table S3. The lack of induction of many of the known immune response genes after 5 hours of exposure to *E. tarda *suggests that at that time, tissue infection has not yet been established.

### Time course analysis of marker gene expression in the immersion system

**Figure 3:**
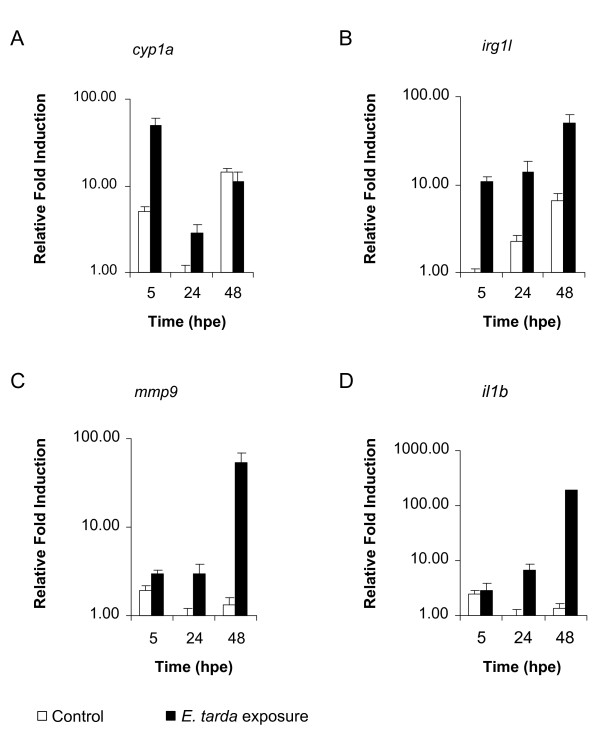
**Time course analysis of marker gene expression in embryos treated by immersion in *E. tarda *suspension**. Embryos were immersed at 25 hpf for 5 h in *E. tarda *suspension or in clean egg water as a control. Subsequently, embryos were washed and transferred to fresh egg water. RNA was isolated from pools of 20 embryos at 5, 24 and 48 h after the start of exposure (hpe) and the expression levels of *cyp1a *(a), *irg1l *(b), *mmp9 *(c), and *il1b *(d) were quantified by qPCR. A representative example of three independent experiments is shown. Relative induction levels are shown with the lowest expression level set at 1.

**Figure 4:**
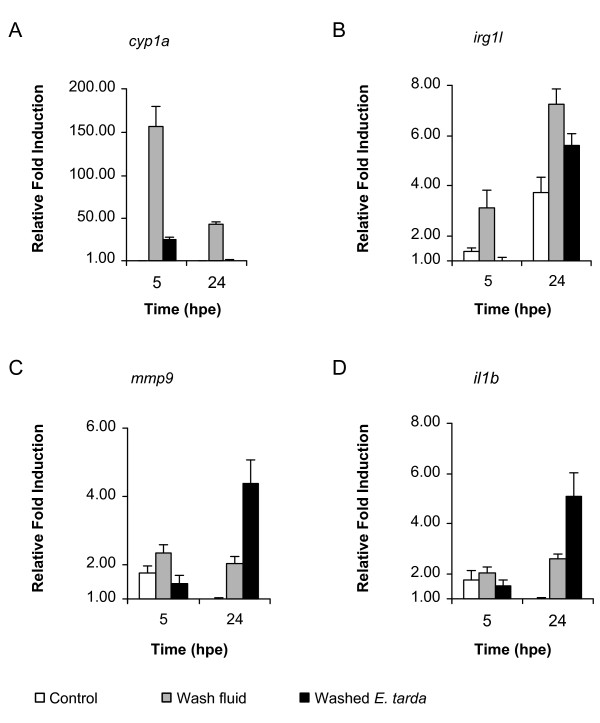
**Marker gene expression in immersion tests after fractionation of *E. tarda *suspension**. The *E. tarda *suspension as used for the immersion experiments in Figure 1-3 was separated into two fractions by centrifugation. Embryos were immersed at 25 hpf for 5 h in the wash fluid obtained after centrifugation, or in the resuspended bacterial pellet (washed bacteria), or in clean egg water as a control. Subsequently, embryos were washed and transferred to fresh egg water. RNA was isolated from pools of 20 embryos at 5 and 24 h after the start of exposure (hpe) and the expression levels of *cyp1a *(a), *irg1l *(b), *mmp9 *(c), and *il1b *(d) were quantified by qPCR. A representative example of two independent experiments is shown. Relative induction levels are shown with the lowest expression level set at 1.

To determine whether a stronger immune response is induced at later time points after exposure to *E. tarda*, we performed a time-course qPCR analysis of several immune related genes. In addition to the putative immune markers *cyp1a*, *irg1l *and *stc1 *found in the microarray analysis (Additional file 1, Table S1), the known immune markers *il1b*, *mmp9 *and *tnfa *were chosen for the time course analysis of the *E. tarda *exposure. Embryos immersed in 10^8 ^CFUs/ml of *E. tarda *were snap-frozen in pools of 20 embryos at 5, 24 and 48 hours post exposure (hpe). RNA was isolated from pools of embryos collected at each time point and the expression of the chosen markers was analyzed. The results showed that *cyp1a *is primarily a marker for the early response towards *E. tarda*, showing 10 times higher expression in *E. tarda*-exposed than in untreated embryos at 5 hpe, but less than 3-fold induction at 24 hpe and no induction at 48 hpe (figure 3a). This might suggest that *cyp1a *induction is the result of an epithelial response. *Irg1l *was induced between 10 and 50 fold at all time points tested (figure 3b). The *mmp9 *(figure 3c) and *il1b *genes (figure 3d) showed little to no induction at 5 hpe, but induction started to increase at 24 hpe and reached 54 to 212-fold induction at 48 hpe. The induction of *tnfa *and *stc1 *was highly variable between the different experiments and therefore excluded in further analyses (data not shown). To test the possibility that the early response in the immersion system might be elicited by cell membrane components or other molecules released by the bacteria, we separated the *E. tarda *suspension used for the immersion experiments into two fractions by centrifugation. Exposure of embryos either to the wash fluid obtained after centrifugation or to the resuspended bacterial pellet, showed that expression of *cyp1a *and *irg1l *was induced to higher levels by the wash fluid than by the washed bacteria, while the opposite was observed for the induction of *il1b *and *mmp9 *(Figure 4). Therefore, the early transcriptional induction of *cyp1a *and *irg1l *appears not to be due to bacterial infection.

### Immune response in single embryos after static immersion in *E. tarda*

The variability in mortality rates in the static immersion system, led us to hypothesize that not all embryos become systemically infected with this method. At 4 days after *E. tarda *immersion, none of the surviving embryos, even those that were close to dying, showed clear fluorescence of the mCherry marker plasmid. Subsequently, we plated individual surviving embryos for CFU counting. From five surviving embryos, of which three showed a slow heart beat indicative of approaching death, we obtained CFU counts of 140 to 690 per individual embryo. In contrast, the egg water medium of these embryos, kept individually in well plates, contained between 80,000 and 300,000 CFUs. It cannot be ascertained from CFU plating if the surviving embryos were actually infected with low numbers of bacteria or that the low CFU counts resulted from bacteria sticking to the surface epithelium of these embryos. However, it is clear that the surviving embryos did not carry heavy infections.

**Figure 5:**
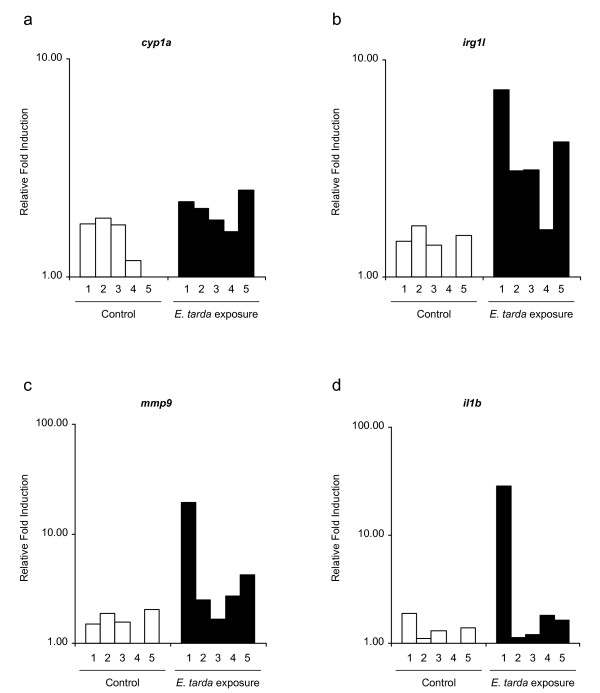
**Marker gene expression in individual embryos treated by immersion in *E. tarda *suspension**. Pools of 20 embryos were immersed at 25 hpf for 5h in *E. tarda *suspension or in clean egg water as a control. Subsequently, embryos were washed and transferred to fresh egg water. RNA was isolated from 5 single embryos at 48 h after the start of exposure (hpe) and expression levels of *cyp1a *(a), *irg1l *(b), *mmp9 *(c) and *il1b *(d) were measured by qPCR. Relative induction levels are shown with the lowest expression level set at 1.

To further test our hypothesis that not all embryos are systemically infected after immersion, we used an RNA-isolation protocol for single embryos [45]. Five single embryos exposed for 5 h to 10^8 ^CFUs/ml of *E. tarda *and five single embryos grown under non-inoculated circumstances were snap-frozen at 48 hpe. RNA was isolated from each embryo and qPCR analysis was done on *cyp1a*, *irg1l, mmp9*, and *il1b *(figure 5). Expression of *cyp1a *showed little to no induction, similar to what we observed in the analysis of pools of embryos at 48 hpe. The difference in induction of *il1b *and *mmp9 *between individual embryos was much more pronounced than we initially expected. Out of the five embryos tested, only one showed a high induction of both markers compared to the control embryos. All embryos showed induction of *irg1l*, but a strong induction of this gene was only observed in the embryo that showed a high *il1b *and *mmp9 *induction, which might indicate that *irg1l *is involved in both an initial response to bacterial components and a later systemic immune response.

### Immune response in single embryos after caudal vein injection of *E. tarda*

**Figure 6:**
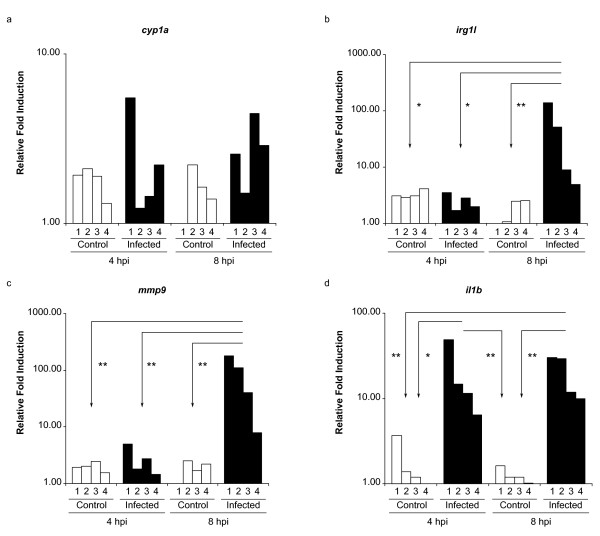
**Marker gene expression in individual embryos in response to injection of *E. tarda***. Expression levels of *cyp1a *(a), *irg1l *(b), *mmp9 *(c) and *il1b *(d) were measured by qPCR in 4 single embryos at 4 h and 8 h after injection (hpi) of approximately 200 CFUs of *E. tarda *into the caudal vein of embryos at 28 hpf. Control embryos were injected with PBS. Relative induction levels are shown with the lowest expression level set at 1. Lines with * indicate a significant difference of P < 0.05. Lines with ** indicate a significant difference of P <0.01 (tested by two-way ANOVA analysis of log-transformed data with the Bonferroni method as post-hoc test).

Results of immersion experiments suggested that induction of *il1b *and *mmp9 *expression may be specifically correlated with systemic infection. To exclude that the large variation in *il1b *and *mmp9 *induction found after immersion might be due to individual variation in responsiveness of different embryos, we decided to compare the immersion system with intravenous infection. Embryos were injected in the caudal vein with 200 CFUs of *E. tarda *at 28 hpf and snap-frozen individually at 4 and 8 hours post infection (hpi) after which RNA was isolated. As before, qPCR analysis was done on *cyp1a*, *irg1l*, *mmp9*, and *il1b *(figure 6). The results show that the genes *irg1l*, *mmp9*, and *il1b *were induced at much higher levels than in the immersion system, whereas *cyp1a *showed similar induction (2-5-fold) as in the immersion system in some embryos or no induction in other embryos. Expression of *il1b *was clearly induced in all embryos at 4 hpi, while *mmp9 *was induced only in two embryos at this time point and *irg1l *was not induced. Although induction of *mmp9 *and *irg1l *at 8 hpi was consistent, the induction levels showed large variation, ranging between 7- and 180-fold for *mmp9 *and between 4- and 140-fold for *irg1l*. Induction levels of *il1b *between individual embryos were the least variable, ranging between 5- and 50-fold at 4 hpi and between 10- and 30-fold at 8 hpi. Compared to injection of 200 CFUs, injection of 25 CFUs resulted in lower *il1b *and *mmp9 *induction levels (Additional file 4, Figure S1). Furthermore, these genes were induced at much higher levels by 200 CFUs of live bacteria than by the same dose of heat-killed bacteria.

**Figure 7:**
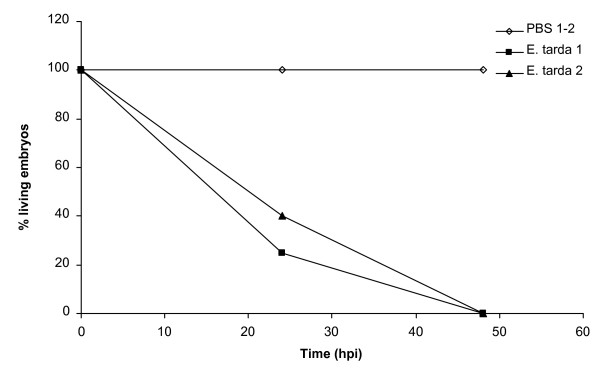
**Survival curve of embryos infected by injection of *E. tarda***. Survival after injection of approximately 200 CFUs of *E. tarda *into the caudal vein of embryos at 28 hpf was monitored for two days, after which no embryos survived. Control embryos were injected with PBS. Experiments were performed in duplicate. In each experiment 20 embryos were used per treatment group.

In addition to the analysis of *cyp1a*, *irg1l*, *mmp9 *and *il1b *induction, we monitored the embryos for two days after injection for appearance of fluorescence from the mCherry-labeled *E. tarda *and for survival. In all injection experiments embryos showed fluorescence at 24 hpi (data not shown) and mortality after injection was very consistent, reaching 100% at 48 hpi (figure 7). Based on these results we conclude that reproducible systemic infection of zebrafish embryos can be achieved by microinjection of *E. tarda *bacteria, accompanied by induction of *il1b *and *mmp9 *expression.

### Microarray analysis of embryos infected by caudal vein injection

Microarray analysis was used to further characterize the immune response in response to microinjection of *E. tarda *bacteria and compare this with the previous microarray results of the immersion system and with our published data of the response to *Salmonella typhimurium *injection [9]. Single infected and mock-injected embryos were analyzed at 8 hpi in triplicate. In gene ontology analysis we observed significant enrichment of the GO-terms "immune system process" and "response to stimulus" (Additional file 5, Table S4), whereas these GO-terms were not enriched in results of the immersion method (Additional file 2, Table S2). In addition, functional annotation using DAVID [46] showed significant enrichment of the KEGG pathways for apoptosis and for Toll-like receptor, adipocytokine, NOD-like receptor, insulin, MAP kinase, RIG-I-like receptor, ErbB, and Jak-Stat signaling. Manual annotation of the induced gene group showed several representatives of the categories complement activation and acute phase response, immune-related transcription factors and signaling components, cytokines and chemokines, apoptosis, and defense response (Additional file 6, Figure S2). In addition, many genes that were not previously linked to the immune response were differentially expressed, including genes involved in signal transduction, transporting activity and metabolism (Additional file 6, Figure S2). Out of 498 significantly regulated probes at 8 hpi (Additional file 7, Table S5), only 2 down-regulated probes (for *vtg6 *and an unannotated transcribed locus) and 1 up-regulated probe (for an unknown gene) were also significantly changed in the immersion system at 5 hpe. The microarray comparison supports that the transcriptional signatures of embryos subjected to immersion and injection are markedly different, although it should be noted that the immersion and injection data are not directly comparable due to a few hours difference in time to respond to the bacteria and in the developmental stage of assessment. The *E. tarda *injection microarray data were also compared with our previous microarray data set of intravenous *Salmonella typhimurium *infection of embryos at 2, 5 and 8 hpi [9]. This comparison showed an overlap of 141 probes with significantly changed expression in response to both pathogens (Additional file 7, Table S5). These probes represented among others *tnfb*, *il1b*, *cxcl-c1c*, *mmp9*, *ncf1*, *mxc*, *pglyrp5*, *hamp1 *and several signal transduction (e.g. *tlr5b*, *irak3*, *nfkbiaa*, *pim1*, *socs1/3a/3b*) and transcription factor genes (e.g. *atf3*, *elf3, fos*, *junb*, *irg9*/*11*, *rel*, *stat1*) (Additional file 7, Table S5).

## Discussion

Zebrafish is being established as an alternative vertebrate model to murine models for infection research. To enable large scale mutant and chemical screening the development of an easily applicable infection test system is highly desired. In this report we studied the effectiveness and variability of treatment of zebrafish embryos by static immersion in *Edwardsiella tarda*, a method previously described by Pressley et al. [32], in comparison with the caudal vein injection method.

In order to perform large scale screenings, a model test system should be optimized for a reproducible response. Our results confirmed the ability of *E. tarda *to cause mortality in zebrafish embryos after static immersion. However, the mortality rate was highly variable between different experiments, ranging from 25 - 75%, comparable to the mortality rate of 31% reported by Pressley *et al *[32]. In order to find a more reproducible readout, we performed microarray analysis on zebrafish embryos that had been exposed to *E. tarda *by static immersion. Surprisingly, only a small number of genes showed differential expression. In contrast, a much larger number of genes were regulated by immersion in bacterial suspensions of *E. coli *and *P. aeruginosa *strains PAO1 and PA14 that do not cause any mortality. In addition, very few immune-related genes were induced by immersion in *E. tarda *and we observed no induction of *il1b *and *tnfa*, which showed transient induction patterns between 2 and 12 hpi in the study of Pressley et al. [32].

Interestingly, *cyp1a *was highly induced by all tested bacteria. In *E. tarda *immersion experiments the induction of this gene preceded that of *il1b *and *mmp9 *induction. Our results suggest that this gene is not induced by direct exposure to the bacteria, but by released cell membrane components or other molecules. Expression of *cyp1a *was most strongly induced by *P. aeruginosa*. *Cyp1a *is known to be induced by toxic chemicals in vascular endothelium, but also in the epithelium of the gills [34,35]. *P. aeruginosa *PAO1 and PA14 are known to secrete large amounts of toxins and protein virulence factors [47-50]. Since *cyp1a *belongs to the cytochrome P450 family, its induction might be involved in a detoxification response. The observation that many of the genes regulated by *P. aeruginosa *are associated with the GO term "response to stress", and the lack of enrichment of genes with the GO-term "immune system process" is consistent with a response to toxins rather than an immune response to systemic infection.

A further time-course analysis by qPCR of pools of embryos subjected to *E. tarda *immersion showed strong induction of *il1b *and *mmp9 *after 48 hours. In addition, the *irg1l *gene, one of the few immune-related genes identified in the microarray study, was also induced at later time points after exposure to *E. tarda*. The *irg1l *gene is homologous to mammalian *irg1*, expression of which in murine macrophages is induced by cytokines, agonists of TLR signalling, and by mycobacterial infections [36-39]. Sequence similarity of *irg1l *and mammalian *irg1 *with bacterial methylcitrate dehydratases suggests an important role in metabolism, but the function in vertebrates remains unknown. When we analyzed gene expression at the level of single embryos we observed that *il1b *and *mmp9 *were highly expressed in only one out of five treated embryos. Expression of *irg1l *was induced in all embryos, but only at a high level in the same embryos that also showed induction of *il1b *and *mmp9*. One possible explanation for the variable results of the static immersion assay is that embryos can individually differ in their resistance towards *E. tarda*. To test this, we compared the immersion system with intravenous injection of bacteria. In contrast to the relatively low and highly variable mortality rates that we observed with the immersion method, injection of bacteria resulted in a reproducible rate of 100% mortality within 2 days. Strong individual differences in levels of gene expression were also observed in the injection system, but nevertheless, induction of the proinflammatory marker genes *il1b *and *mmp9 *was positive in all embryos and their induction levels correlated with the dose of live bacteria injected. Furthermore, microarray experiments with single injected embryos showed a consistent profile of strong activation of proinflammatory and defense genes and regulatory genes of the immune response. The observed gene expression profiles are concordant with those observed for intravenous *Salmonella typhimurium *infection of embryos at similar time periods after injection [9]. Detailed comparisons of the responses to *E. tarda *and *S. typhimurium *infections will be part of a follow-up study that will also address the function of essential immune regulators in these models.

Since only a subset of embryos in the immersion assay showed induction of immune response markers and mortality it is conceivable that only these embryos were systemically invaded by *E. tarda *bacteria or that non-responsive embryos were invaded by a much lower number of bacteria. Neither fluorescence monitoring nor CFU plating indicated that embryos become heavily infected close before dying. On the contrary, bacteria were present in high abundance in the egg water medium and only few were associated with dying embryos. It therefore remains uncertain whether infection or toxic insult is the actual cause of mortality in the immersion system. It is possible that the variable immune gene inductions and mortality rates resulted from slight epithelial damage to embryos that occurred during dechorionating and washing procedures, providing sites of entry for bacteria. Instead of exposure at 1 dpf, we used the same immersion protocol on embryos of 3 dpf, which is the developmental stage when the mouth opens and the gut begins to be colonized by environmental bacteria [51]. We followed survival until 5 dpf, which is the time-point up to which larvae do not fall under the European animal experimentation law, but did not observe mortality within that time (data not shown).

Besides being more practical for high-throughput screening, an immersion system might be preferred as a more natural route of infection compared to injection methods. However, we conclude that the *E. tarda *immersion method as applied here on 1-day-old zebrafish embryos is not suitable to achieve reproducible systemic infection. Therefore, unless a more virulent strain can be identified, injection remains the preferred method of infection for screening purposes. On the other hand, the immersion system is shown to be useful for studying epithelial or other tissue responses towards cell membrane or other molecules that are shed or released by bacteria. An alternative solution for high-throughput screening of systemic infection is the use of robotic yolk injection system recently developed for *Mycobacterium marinum *infection [52]. However, the wild type *E. tarda *FL6-60 strain used here causes early lethality after yolk injection (data not shown). The use of less virulent (wild type or mutant) strains might provide a solution for this problem. In any case, our gene expression profiling data sets will be necessary for comparisons to the immune response in such alternative yolk infection methods.

## Conclusions

Zebrafish embryos proved to be remarkably resistant to becoming systemically infected after immersion in bacterial suspensions of *E. tarda*, whereas they are strongly susceptible to intravenous injection of this pathogen. While the microarray expression profile of intravenously infected embryos indicates a strong inflammatory response, the transcriptional signature of embryos subjected to immersion was markedly different. Our data suggest that most of the early transcriptional responses in the immersion system may reflect an epithelial or other tissue response towards cell membrane or other molecules that are shed or released by bacteria. Therefore, our studies on the expression analysis in the bacterial immersion system will be useful for future analysis of signal transduction pathways underlying responses to external bacteria and secreted putative virulence factors and toxins. Transient induction of the cytochrome P450 gene *cyp1a *was specifically observed in immersion experiments but not when embryos were systemically infected by injection. In addition, our identification of the *irg1l *gene as a rapid response factor to externally added bacteria deserves further study of the underlying signal transduction pathway as compared to systemic tissue responses. Although *irg1l *is also up-regulated during systemic infection, its expression kinetics in embryos immersed in *E. tarda *is very different from that of well-known inflammation genes such as *il1b *and *mmp9*. Considering the important function of epithelial cells in cross talk with cells of the innate immune system, as recently underscored by studies in zebrafish [31], further analysis of infection modes using the identified marker genes will help to better understand the systemic response of tissues toward an infection in a whole organism context.

## Methods

### Zebrafish husbandry

Zebrafish were handled in compliance with the local animal welfare regulations and maintained according to standard protocols (zfin.org). An albino strain was used for all immersion and injection experiment, except for the microarray study of injected embryos that was performed with wild type zebrafish. Embryos were grown at 28.5-30°C in egg water (60 μg/ml Instant Ocean salts). For the duration of bacterial injections embryos were kept under anesthesia in egg water containing 0.02% buffered 3-aminobezoic acid ethyl ester (tricaine; Sigma-Aldrich).

### Bacterial immersion and injection experiments

*Edwardsiella tarda *strain FL6-60 obtained from Dr. P. Klesius (USDA, Auburn, AL) is the identical strain as used in the study of Pressley et al. [32]. Identity of this strain was confirmed by performing nucleotide sequencing of the entire genome using Illumina technology with a 180-fold coverage (Genbank accessions CP002154 and CP002155). FL6-60 was grown over night on tryptic soy agar (Difco) at 28°C and subsequently a liquid culture in tryptic soy broth (TSB, Difco) was inoculated and grown overnight at 28°C with shaking at 150 rpm. *Pseudomonas aeruginosa *PAO1 and PA14 and *Escherichia coli *were grown over night in Luria-Bertani broth (LB) [53] at 37°C. For immersion experiments bacterial cultures were centrifuged in 50 ml tubes and the pellet was subsequently suspended in egg water to a final 10^8 ^CFU/ml for *E. tarda *and *E. coli*, and 10^9 ^CFU/ml for *P. aeruginosa*. Embryos were dechorionated at 24 hpf by a 3-5 min pronase treatment (2 mg/ml in embryo medium prewarmed to 30°C) and left to recover for one hour in egg water. Subsequently pools of 20 embryos in 6-well plates were immersed in 5 ml of the bacterial suspension and incubated for 5 hours at 28°C. After 5 hours of incubation, the embryos were either snap-frozen in liquid nitrogen or transferred to a new 6-wells plate, washed 3 times in egg water, and incubated at 28°C in 5 ml of egg-water. For CFU plating experiments, embryos were kept individually in 2.5 ml of egg water in 24-well plates.

For caudal vein injection experiments, *E. tarda *labeled with mCherry [54] was washed and subsequently suspended in PBS (phosphate-buffered saline) to a final 10^8 ^CFU/ml. Embryos were manually dechorionated at 24 hpf. Approximately 200 CFUs of *E. tarda *were injected into the blood island after the onset of blood flow at 28 hpf, or PBS was injected as a control. After injection, embryos were kept at 28°C and snap-frozen in liquid nitrogen at the required time points.

### RNA isolation from pools of embryos

Pools of 20 - 30 embryos were snap-frozen in liquid nitrogen and subsequently stored at -80°C. Embryos were homogenized in 1 ml of TRI reagent (Ambion), and subsequently total RNA was extracted according to the manufacturer's instructions. The RNA samples were incubated for 20 min at 37°C with 10 U of DNaseI (Roche Applied Science) to remove residual genomic DNA before purification using the RNeasy MinElute Cleanup kit (Qiagen) according to the RNA clean-up protocol. The integrity of the RNA was confirmed by lab-on-chip analysis using the 2100 Bioanalyzer (Agilent Technologies). Samples used for microarray analysis had an average RNA integrity number value of 9 and a minimum RNA integrity number value of 8.

### RNA isolation from single embryos

The single embryo RNA isolation procedure was performed according to de Jong *et al. *[45]. Embryos were individually snap-frozen in liquid nitrogen and subsequently stored at -80°C. A frozen embryo was crushed with a chilled pestle and homogenized in 300 μl of TRI reagent (Ambion). 60 μl of chloroform was added and the mixture was transferred to a 1.5 ml reaction tube containing 50 mg phase lock gel (Eppendorf) and incubated at room temperature for 5 minutes. The mixture was centrifuged at 12000 g at 4°C for 15 minutes, after which the aqueous phase was transferred to a fresh tube. 1 volume of 70% ethanol was added and the mixture transferred to a RNeasy MinElute Cleanup kit (Qiagen) column which was centrifuged 15 seconds at 8000 g. 500 μl RPE buffer from the kit was applied to the column and centrifuged 15 seconds at 8000 g. 500 μl 80% ethanol was applied to the column and centrifuged 2 minutes at 8000 g. The collection tube was replaced and the column centrifuged 5 minutes at 14000 g. 14 μl H_2_O was applied to the column and centrifuged 1 minute at 14000 g. The average amount of RNA isolated from a single embryo was 500 ng.

### Microarray analysis

The microarray slides were custom-designed by Agilent Technologies as previously described [9]. The slides contained in total 43,371 probes of a 60-oligonucleotide length.

Amino-allyl-modified amplified RNA (aRNA) was synthesized in one amplification round from total RNA using the Amino Allyl MessageAmp II aRNA Amplification kit (Ambion). The amount of total RNA used was 1 μg in experiments using RNA from pooled embryos and 400 ng in experiments using RNA from single embryos. Subsequently, 6 μg of amino-allyl-modified aRNA was used for coupling of monoreactive Cy3 and Cy5 dyes (GE Healthcare) and column purified. Samples from embryos immersed in *E. tarda*, *E. coli*, or *P. aeruginosa *suspensions or untreated control embryos were labeled with Cy5 and hybridized against a Cy3-labeled common reference that consisted of a mixture of all samples from the immersion experiments. *E. tarda *and control immersions were analyzed in triplicate using pools of 20 embryos and compared with single experiments of *E. coli*, *P. aeruginosa *PAO1 and *P. aeruginosa *PA14 immersion. For the *E. tarda *injection study, infected embryos and control embryos injected with the PVP-carrier solution were labeled with Cy5 and analyzed in triplicate against a Cy3-labeled common reference. Dual-color hybridization of the microarray chips was performed at ServiceXS according to Agilent protocol G4140-90050 version 5.7 (http://www.Agilent.com) for two-color microarray-based gene expression analysis.

Microarray data were processed from raw data image files with Feature Extraction Software 9.5.3 (Agilent Technologies). Processed data were subsequently imported into Rosetta Resolver 7.0 (Rosetta Biosoftware) and subjected to default ratio error modeling. The raw data were submitted to the Gene Expression Omnibus database (http://www.ncbi.nlm.nih.gov/geo) under accession no. GSE28486. To compare samples from treatment groups to the control samples re-ratio analyses were performed using the Rosetta built-in re-ratio with common reference application. Data were analyzed at the level of UniGene clusters (UniGene build no. 105) and at probe level. Significance cut-offs for the ratios were set at 1.5-fold change at *P *< 10^-4 ^for analysis at UniGene cluster level and *P *< 10^-5 ^for analysis at probe level.

Gene ontology (GO) analysis was performed using the GeneTools eGOn v2.0 web-based gene ontology analysis software (http://www.genetools.microarray.ntnu.no) [55]. KEGG pathway analysis was performed using DAVID tools for functional annotation (http://david.abcc.ncifcrf.gov/) [46]. In addition, genes were manually annotated based on information in the ZFIN (zfin.org) and NCBI Entrez Gene databases, and PubMed abstracts.

### cDNA synthesis and quantitative reverse transcriptase PCR

For RNA samples from pooled embryos, cDNA synthesis reactions were performed in a 20 μl mixture of 500 ng of RNA, 4 μl of 5x iScript reaction mix (Bio-Rad Laboratories), and 1 μl of iScript reverse transcriptase (Bio-Rad Laboratories). For RNA samples from single embryos, cDNA synthesis reactions were performed in a 10 μl mixture of 100 ng of RNA, 2 μl of 5x iScript reaction mix (Bio-Rad Laboratories), and 0.5 μl of iScript reverse transcriptase (Bio-Rad Laboratories). The reaction mixtures were incubated at 25°C for 5 min, 42°C for 30 min, and 85°C for 5 min.

Real-time PCR was performed using the Chromo4 Real-time PCR detection system (Bio-Rad Laboratories) according to the manufacturer's instructions. Each reaction was performed in a 25-μl volume comprised of 1 μl of cDNA, 12.5 μl of 2x iQ SYBR Green Supermix (Bio-Rad Laboratories), and 10 pmol of each primer. Cycling parameters were 95°C for 3 min to activate the polymerase, followed by 40 cycles of 95°C for 15 s and 59°C for 45 s. Fluorescence measurements were taken at the end of each cycle. Melting curve analysis was performed to verify that no primer dimers were amplified. All reactions were performed as technical duplicates. For normalization, peptidylprolyl isomerase A-like (*ppial*), which showed no changes over the infection time course series, was taken as reference. Results were analyzed using the ΔΔ*C*_*t *_method. Sequences of forward and reverse primers are described in Additional file 8, Table S6.

## Competing interests

The authors declare that they have no competing interests.

## Authors' contributions

JJvS designed and conducted experiments, analyzed the data, and wrote the manuscript. OWS and AO contributed to the experiments and data analysis. GVB, HPS and AHM conceived and supervised the study, and edited the manuscript. All authors read and approved the final version.

## Supplementary Material

Additional file 1 **Genes up-regulated in zebrafish embryos at 5 h after immersion in *E. tarda, E. coli*, *P. aeruginosa *PAO1, and *P. aeruginosa *PA14**. The table lists the genes that had a significant (P < 0.0001) signature at 5 h after immersion of zebrafish embryos at 25 hpf in *E. tarda*, *E. coli*, *P. aeruginosa *PAO1 or *P. aeruginosa *PA14 suspension. Click here for file

Additional file 2 **Gene ontology analysis of the up-regulated genes in zebrafish embryos after immersion in *E. tarda*, *E. coli*, *P. aeruginosa *PAO1, and *P. aeruginosa *PA14**. Genes up-regulated significantly (p < 0.0001, no fold change cut-off) after immersion in *E. tarda*, *E. coli*, *P. aeruginosa *PAO1, and *P. aeruginosa *PA14 were subjected to a master-target statistical test using the web-based eGOn software. The table indicates the number of genes associated with the indicated GO categories for the master (i.e. all genes on the microarray) and targets (i.e. the differentially expressed gene lists of the treatment groups). Yellow indicates significant enrichment of GO-categories in the treatment groups, and blue indicates significant underrepresentation (P < 0.05) Click here for file

Additional file 3 **Up-regulated genes with the GO-term "response to stimulus" after immersion in *E. tarda*, *E. coli*, *P. aeruginosa *PAO1, and *P. aeruginosa *PA14**. Gene ontology analysis using eGOn identified 5, 22, 41 and 61genes (indicated with +) that were up-regulated (P < 0.0001) after immersion of zebrafish embryos in *E. tarda*, *E. coli*, *P. aeruginosa *PAO1, or *P. aeruginosa *PA14, and that were associated with the GO-term 'response to stimulus'. Click here for file

Additional file 4 **Marker gene expression in individual embryos in response to injection of different doses of live and heat-killed *E. tarda***. Expression levels of *mmp9 *(a) and *il1b *(b) were measured by qPCR in single embryos at 8 h after injection (hpi) of approximately 25 or 200 CFUs of live or heat-killed (45 min at 95°C) *E. tarda *into the caudal vein embryos at 28 hpf. Control embryos were injected with PBS. Relative induction levels are shown with the lowest expression level set at 1. Lines with * indicate a significant difference of P < 0.05 (tested by one-way ANOVA analysis with the Bonferroni method as post-hoc test). Click here for file

Additional file 5 **Gene ontology analysis of the up-regulated genes in zebrafish embryos injected with *E. tarda***. Genes up-regulated significantly up-regulated (P < 0.00001) after injection of zebrafish embryos with *E. tarda *were subjected to a master-target statistical test using the web-based eGOn software. The table indicates the number of genes associated with the indicated GO categories for the master (i.e. all genes on the microarray) and the target (i.e. differentially expressed genes after *E. tarda *injection). Yellow indicates significant enrichment of GO-categories in the treatment groups, and blue indicates significant underrepresentation (P < 0.05) Click here for file

Additional file 6 **Heat-map and annotations of genes differentially expressed at 8 h after injection of *E. tarda***. Genes were manually annotated and assigned to functional groups based on GO annotations of the zebrafish genes and their human homologues and on searching of PubMed abstracts. (a) Genes previously implicated to be involved in the immune response or novel genes with strong sequence similarity to those genes, (b) genes with known or predicted functions not previously linked to the immune response. Up-regulation is indicated by increasingly bright shades of yellow and down-regulation by increasingly bright shades of blue. The significance cut-off for the analysis was set at P < 0.00001. Click here for file

Additional file 7 **Genes showing differential expression in zebrafish embryos at 8 h after injection of *E. tarda***. The table lists the probes that had a significant (P < 0.00001) signature at 8 h after injection of 200 CFU of *E. tarda *into the caudal vein of zebrafish embryos at 28 hpf. Genes were manually annotated and assigned to functional groups based on GO annotations of the zebrafish genes and their human homologues and on searching of PubMed abstracts. Genes are divided into 3 categories: 1: annotated genes previously implicated in the vertebrate immune response and novel/hypothetical genes with sequence similarity to these immune response genes; 2: annotated or novel/hypothetical genes whose known or predicted functions have not been linked to the vertebrate immune response; 3: genes with unknown function. Genes from categories 1 and 2 are ordered by functional annotation groups. For comparison fold change and P-values are shown of probes that also had a significant signature in the *E. tarda *immersion microarray or in previous microarray data of *S. typhimurium *injection at 2, 5 and 8 hpi [9]. Click here for file

Additional file 8 **qPCR primer sequences**. Primer sequences and Genbank accessions for genes analyzed in this study. Click here for file
