# Supplementary figures and images for: Comparison of static immersion and intravenous injection systems for exposure of zebrafish embryos to the natural pathogen Edwardsiella tarda
Source: BMC Immunol. 2011 Oct 17;12:58. doi: 10.1186/1471-2172-12-58 (PMC3206475; doi:10.1186/1471-2172-12-58)

*il1b*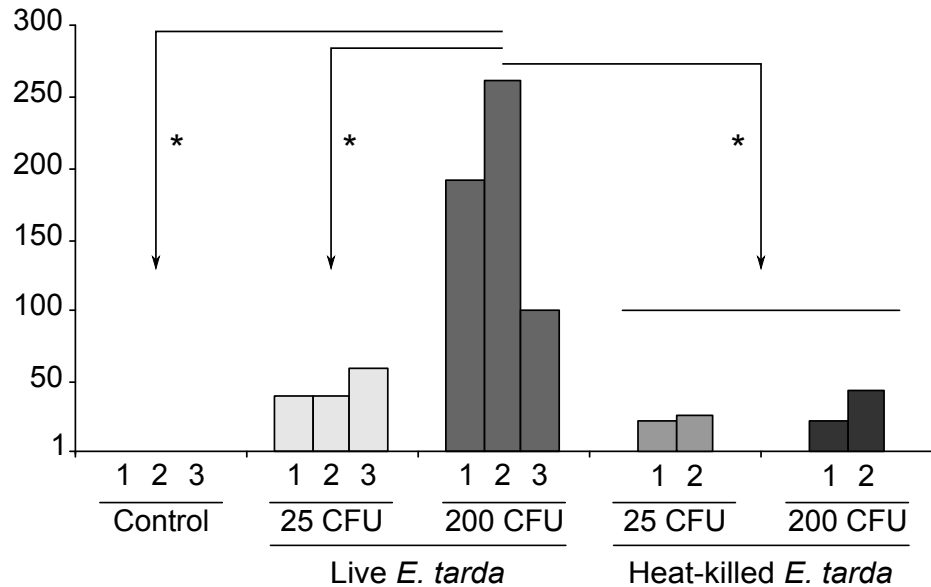*mmp9*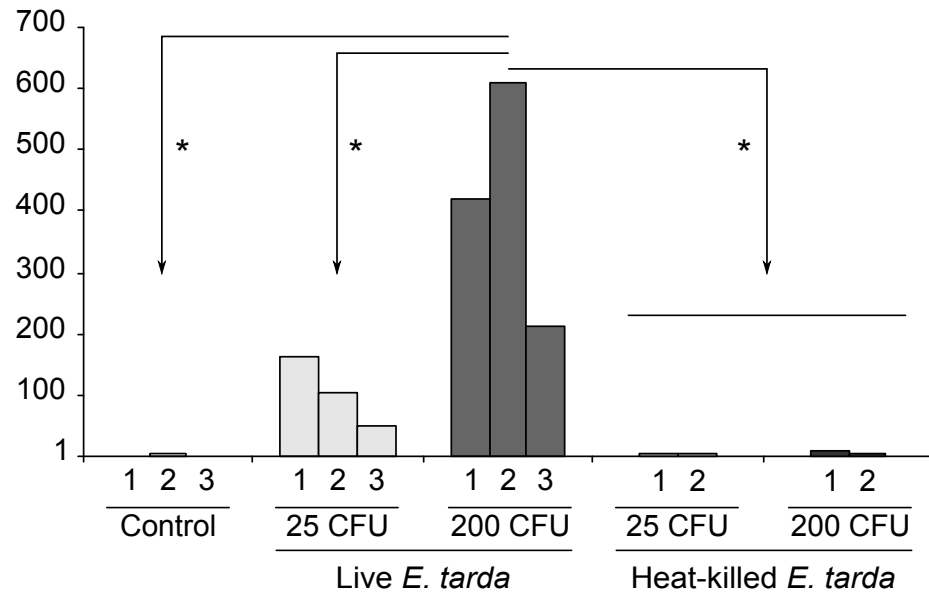

Supplement: Additional file 4 — Marker gene expression in individual embryos in response to injection of different doses of live and heat-killed E. tarda. Expression levels of mmp9 (a) and il1b (b) were measured by qPCR in single embryos at 8 h after injection (hpi) of approximately 25 or 200 CFUs of live or heat-killed (45 min at 95°C) E. tarda into the caudal vein embryos at 28 hpf. Control embryos were injected with PBS. Relative induction levels are shown with the lowest expression level set at 1. Lines with * indicate a significant difference of P < 0.05 (tested by one-way ANOVA analysis with the Bonferroni method as post-hoc test). [file 1471-2172-12-58-S4.PDF]
