# Supplementary material for: Comparison of static immersion and intravenous injection systems for exposure of zebrafish embryos to the natural pathogen Edwardsiella tarda
Source: BMC Immunol. 2011 Oct 17;12:58. doi: 10.1186/1471-2172-12-58 (PMC3206475; doi:10.1186/1471-2172-12-58)
Supplement: Additional file 6 — Heat-map and annotations of genes differentially expressed at 8 h after injection of E. tarda. Genes were manually annotated and assigned to functional groups based on GO annotations of the zebrafish genes and their human homologues and on searching of PubMed abstracts. (a) Genes previously implicated to be involved in the immune response or novel genes with strong sequence similarity to those genes, (b) genes with known or predicted functions not previously linked to the immune response. Up-regulation is indicated by increasingly bright shades of yellow and down-regulation by increasingly bright shades of blue. The significance cut-off for the analysis was set at P < 0.00001. [file 1471-2172-12-58-S6.PDF]

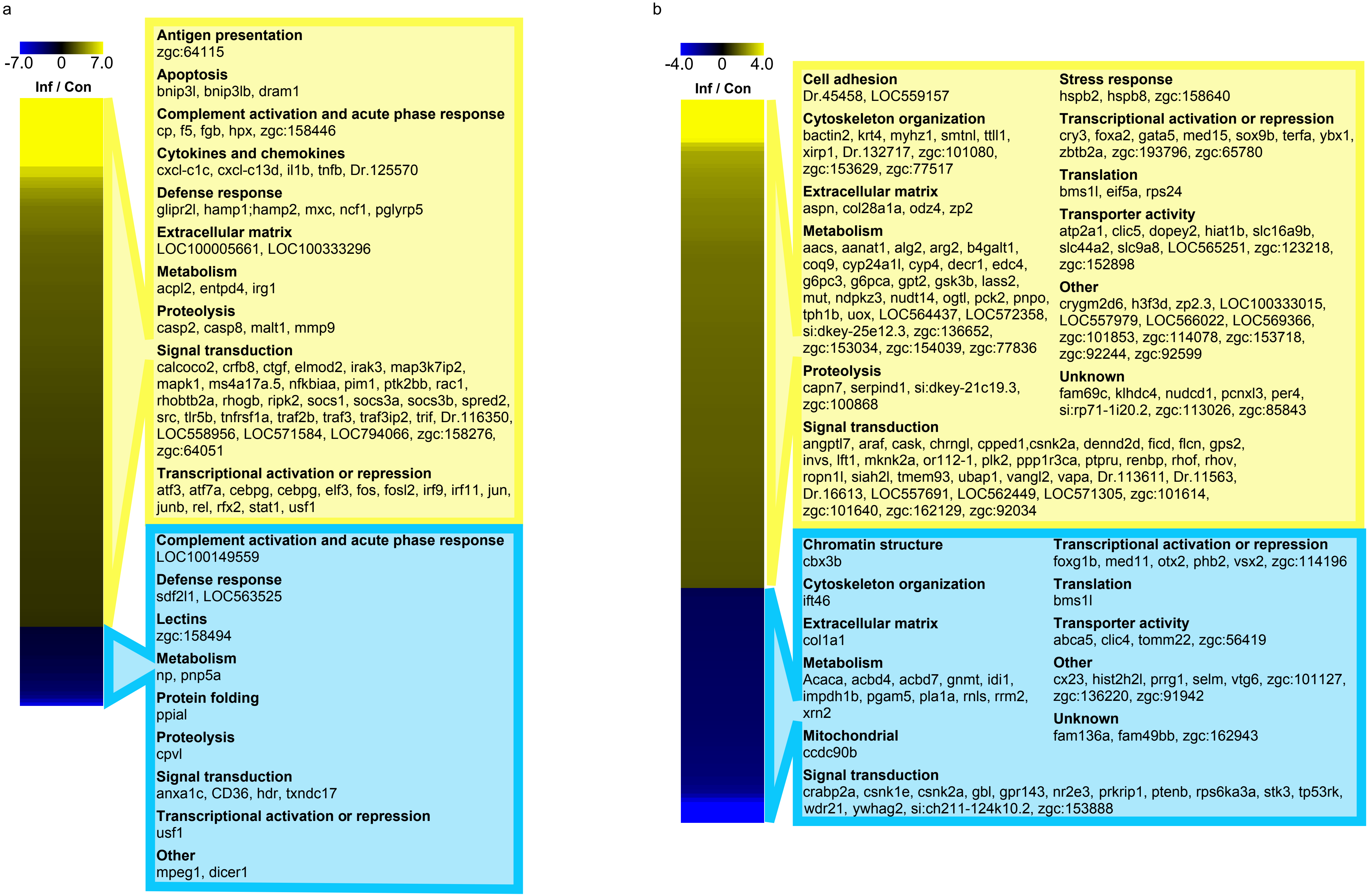
a
-7.0 0 7.0
Inf / Con
Antigen presentation
zgc:64115
Apoptosis
bnip3l, bnip3lb, dram1
Complement activation and acute phase response
cp, f5, fgb, hpx, zgc:158446
Cytokines and chemokines
cxcl-c1c, cxcl-c13d, il1b, tnfb, Dr.125570
Defense response
glipr2l, hamp1;hamp2, mxc, ncf1, pglyrp5
Extracellular matrix
LOC100005661, LOC100333296
Metabolism
acpl2, entpd4, irg1
Proteolysis
casp2, casp8, malt1, mmp9
Signal transduction
calcoco2, crfb8, ctgf, elmod2, irak3, map3k7ip2, mapk1, ms4a17a.5, nfkbiaa, pim1, ptk2bb, rac1, rhobtb2a, rhogb, ripk2, socs1, socs3a, socs3b, spred2, src, tlr5b, tnfrsf1a, traf2b, traf3, traf3ip2, trif, Dr.116350, LOC558956, LOC571584, LOC794066, zgc:158276, zgc:64051
Transcriptional activation or repression
atf3, atf7a, cebpg, cebpg, elf3, fos, fosl2, irf9, irf11, jun, junb, rel, rfx2, stat1, usf1
Complement activation and acute phase response
LOC100149559
Defense response
sdf2l1, LOC563525
Lectins
zgc:158494
Metabolism
np, pnp5a
Protein folding
ppial
Proteolysis
cpvl
Signal transduction
anxa1c, CD36, hdr, txndc17
Transcriptional activation or repression
usf1
Other
mpeg1, dicer1
b
-4.0 0 4.0
Inf / Con
Cell adhesion
Dr.45458, LOC559157
Cytoskeleton organization
bactin2, krt4, myhz1, smtnl, ttll1, xirp1, Dr.132717, zgc:101080, zgc:153629, zgc:77517
Extracellular matrix
aspn, col28a1a, odz4, zp2
Metabolism
aacs, aanat1, alg2, arg2, b4galt1, coq9, cyp24a1l, cyp4, decr1, edc4, g6pc3, g6pca, gpt2, gsk3b, lass2, mut, ndpkz3, nudt14, ogtl, pck2, pnpo, tph1b, uox, LOC564437, LOC572358, si:dkey-25e12.3, zgc:136652, zgc:153034, zgc:154039, zgc:77836
Proteolysis
capn7, serpind1, si:dkey-21c19.3, zgc:100868
Stress response
hspb2, hspb8, zgc:158640
Transcriptional activation or repression
cry3, foxa2, gata5, med15, sox9b, terfa, ybx1, zbtb2a, zgc:193796, zgc:65780
Translation
bms1l, eif5a, rps24
Transporter activity
atp2a1, clic5, dopey2, hiat1b, slc16a9b, slc44a2, slc9a8, LOC565251, zgc:123218, zgc:152898
Other
crygm2d6, h3f3d, zp2.3, LOC100333015, LOC557979, LOC566022, LOC569366, zgc:101853, zgc:114078, zgc:153718, zgc:92244, zgc:92599
Unknown
fam69c, klhdc4, nudcd1, pcnxl3, per4, si:rp71-1i20.2, zgc:113026, zgc:85843
Signal transduction
angptl7, araf, cask, chrngl, cpped1,csnk2a, dennd2d, ficd, flcn, gps2, invs, lft1, mknk2a, or112-1, plk2, ppp1r3ca, ptpru, renbp, rhof, rhov, ropn1l, siah2l, tmem93, ubap1, vangl2, vapa, Dr.113611, Dr.11563, Dr.16613, LOC557691, LOC562449, LOC571305, zgc:101614, zgc:101640, zgc:162129, zgc:92034
Chromatin structure
cbx3b
Cytoskeleton organization
ift46
Extracellular matrix
col1a1
Metabolism
Acaca, acbd4, acbd7, gnmt, idi1, impdh1b, pgam5, pla1a, rnls, rrm2, xrn2
Mitochondrial
ccdc90b
Transcriptional activation or repression
foxg1b, med11, otx2, phb2, vsx2, zgc:114196
Translation
bms1l
Transporter activity
abca5, clic4, tomm22, zgc:56419
Other
cx23, hist2h2l, prrg1, selm, vtg6, zgc:101127, zgc:136220, zgc:91942
Unknown
fam136a, fam49bb, zgc:162943
Signal transduction
crabp2a, csnk1e, csnk2a, gbl, gpr143, nr2e3, prkrip1, ptenb, rps6ka3a, stk3, tp53rk, wdr21, ywhag2, si:ch211-124k10.2, zgc:153888
